# Recurrent Extradural Myxopapillary Ependymoma With Oligometastatic Spread

**DOI:** 10.3389/fonc.2019.01322

**Published:** 2019-11-28

**Authors:** Kristen A. Batich, Richard F. Riedel, John P. Kirkpatrick, Betty C. Tong, William C. Eward, Char Loo Tan, Patricia D. Pittman, Roger E. McLendon, Katherine B. Peters

**Affiliations:** ^1^Department of Medicine, Duke University Health System, Durham, NC, United States; ^2^Division of Medical Oncology, Department of Medicine, Duke University Health System, Durham, NC, United States; ^3^The Preston Robert Tisch Brain Tumor Center, Duke University Health System, Durham, NC, United States; ^4^Duke Cancer Institute, Duke University Health System, Durham, NC, United States; ^5^Department of Radiation Oncology, Duke University Health System, Durham, NC, United States; ^6^Department of Neurosurgery, Duke University Health System, Durham, NC, United States; ^7^Division of Cardiovascular and Thoracic Surgery, Department of Surgery, Duke University Health System, Durham, NC, United States; ^8^Department of Orthopaedic Surgery, Duke University Health System, Durham, NC, United States; ^9^Department of Pathology, Duke University Health System, Durham, NC, United States; ^10^Department of Pathology, National University Health System, Singapore, Singapore

**Keywords:** myxopapillary ependymoma, extradural, glial fibrillary acidic protein, oligometastases, post-sacral

## Abstract

Myxopapillary ependymomas are a slow-growing, grade I type glial tumor in the lumbosacral region. More rarely, they can present as extradural, subcutaneous sacrococcygeal, or perisacral masses, and it is under these circumstances that they are more likely to spread. Here, we report the presentation of a sacrococcygeal mass in patient that was initially resected confirming extradural myxopapillary ependymoma. At initial resection, multiple small pulmonary nodules were detected. This mass recurred 2 years later at the resection site with an interval increase in the previously imaged pulmonary nodules. Resection of both the post-sacral mass and largest lung metastasis confirmed recurrent myxopapillary ependymoma with oligometastatic spread. Because these tumors are rare, with extradural presentation being even more infrequent, to this date there are no definitive therapeutic guidelines for initial treatment and continued surveillance. For myxopapillary ependymoma, current standard of care is first-line maximal surgical resection with or without postoperative radiotherapy depending on the extent of disease and extent of resection. However, there remains insufficient evidence on the role of radiotherapy to oligometastatic foci in providing any further survival benefit or extending time to recurrence. Thus, prospective studies assessing the role of upfront treatment of oligometastases with local resection and adjuvant radiotherapy are needed for improved understanding of extradural myxopapillary ependymoma.

## Background

Myxopapillary ependymomas (MPE) are a rare, slow-growing, well-circumscribed grade I ependymoma (1). They are distinct from grade II (ependymoma) and grade III (anaplastic) tumors by their site of origin and growth rate (2). They most commonly present in younger adults in their third or fourth decade, with a mean age of 36 years at diagnosis, and arise more commonly in men (3, 4). These tumors almost exclusively occur near the conus medullaris, cauda equina, and filum terminale of the spinal cord, but occasionally affect the cervical thoracic spine, intraventricular space, and brain parenchyma. If intradural, these tumors can bleed into the CSF resulting in local spread to these other regions within the CNS (5, 6). More rarely, they can present as extradural, subcutaneous sacrococcygeal, or perisacral masses, and it is under these circumstances that they are more likely to spread systemically in a hematogenous or lymphatic fashion (7–11). Clinically, an extradural lesion is often misdiagnosed as a pilonidal sinus or cyst, but with any soft tissue lesion, the differential must also include sacrococcygeal teratoma, neurogenic tumor, soft tissue sarcoma, metastatic carcinoma, and MPE (12).

Magnetic resonance imaging (MRI) remains the preferred imaging modality of choice, as it identifies the tumor and its relation to the surrounding soft tissues and, if intradural, the terminal spinal cord elements. MRI is also best suited for detection of leptomeningeal spread along the spinal axis (13). Management of ependymoma depends on the tumor grade and location. For spinal cord ependymoma more often presenting in adults, the current standard of care is maximal surgical resection with postoperative radiotherapy (RT) in cases of subtotal resection (14–18). Initial resection and RT has also been employed for MPE. The upfront use of adjuvant radiation is particularly critical when treating extradural MPE that already have a high proclivity to spread systemically (19–22).

Here we report the initial presentation of a sacrococcygeal mass in a young patient. This mass was found to be an extradural MPE. Given the rarity of MPE, with extradural presentation being even more infrequent, there is currently limited understanding of the best measures to control disease and to prevent recurrence. The initial management of extradural MPE, surveillance, and treatment strategy at the time of recurrence are discussed herein.

## Case Report

A previously healthy, active 30 year-old Caucasian male presented with fatigue and dyspnea on exertion and was found to have a pulmonary embolism. The patient was a rare smoker with no previously known risk factors. A thorough oncologic history revealed that he had been living with a small, asymptomatic “bump” on his lower coccyx that had been stable for years. It was first noted to appear shortly after falling from the bed as a small child. At the time of presentation, the patient denied any back pain, discomfort, gait instability, or changes in bowel or bladder function.

**Figure 1:**
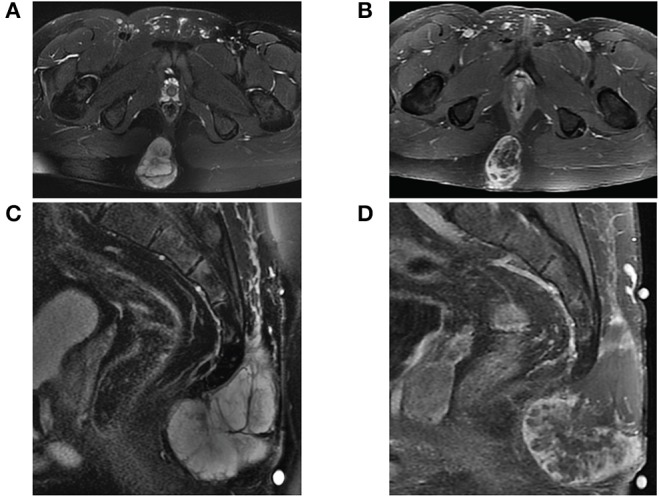
MRI of sacrococcygeal mass prior to initial resection. **(A)** Axial view of fat-saturated T2-weighted and **(B)** contrast-enhanced T1-weighted sequences demonstrate a lobulated expansile mass confined to the soft tissues affixed between the gluteal muscles. The mass on sagittal view is **(C)** T2 hyperintense well-encapsulated within the post-sacral soft tissues without invasion into the sacrococcygeal space and **(D)** heterogeneously enhancing with central necrosis.

**Figure 2:**
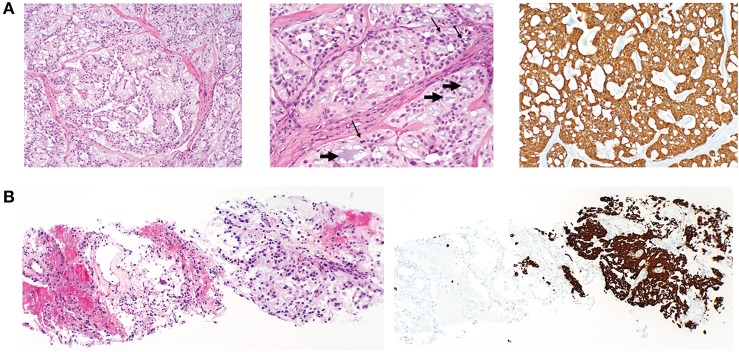
Histopathology of primary postsacral mass and lung metastasis. **(A)** Left: photomicrographs of initially resected postsacral mass demonstrates tumor composed of papillary structures lined by cuboidal glial cells with an intermediate layer of myxoid stroma (original magnification, ×100). Middle: numerous mucin-rich microcysts (short arrows) and mitotic figures (thin arrows) (original magnification ×200). Right: tumor cells are diffusely positive for GFAP (original magnification, ×100). **(B)** Initial lung biopsy of the right lower lobe nodule (8 mm) demonstrates a metastatic mucin-rich tumor (left photo) with adjacent non-neoplastic lung and (right) metastatic focus demonstrating diffuse reactivity with GFAP (original magnification ×100).

The mass was fine needle biopsied showing a myxoid and epithelioid population, thought to be a low-grade sarcoma. The patient then continued on 8 months of oral anticoagulation until the mass was ultimately resected. Pelvic imaging of the asymptomatic mass prior to initial resection revealed an expansile, non-eroding mass in the post-sacral soft tissues (Figure 1). Pathology of the initial resection from the outside hospital was corroborated with secondary review by two board-certified neuropathologists. Microscopic examination revealed focal involvement of the anterior resection margin, with extension in other dimensions to < 1 mm from the remaining operative margins. The 12.1 cm perisacral soft tissue mass displayed tumor composed of papillary structures with central hyalinized blood cells surrounded by cuboidal to elongated tumor cells with an intermediate layer of myxoid stroma (Figure 2A, left). A more solid architecture was also evident, punctuated by scattered mucin-filled microcysts. Focally, few mitotic figures were identified (Figure 2A, middle). The tumor cells showed diffuse and strong immunoreactivity for glial fibrillary acidic protein (GFAP) (Figure 2A, right) and patchy immunoreactivity for pan-cytokeratin and epithelial membrane antigen (EMA) (not shown). Immunohistochemistry for CDX2 was negative. The mass was consistent with MPE (WHO grade I). Retrospective next-generation sequencing was performed on the original perisacral specimen. The original tumor exhibited a low tumor mutational burden (3 mutations/Mb) and was microsatellite stable with an identified truncation of *CTNNA1*, a gene that codes for alpha-catenin of the cadherin family. No other clinically significant alterations or rearrangements were identified.

Extensive imaging at the time of resection additionally revealed small indeterminate bilateral pulmonary nodules in the right and left lower lobes, with one right lower lobe nodule measuring 4 mm in maximum diameter. Pelvic imaging was negative for significant lymphadenopathy but did show visible iliac nodes. No adjuvant therapy was initiated, and the patient was followed with surveillance CT scans.

**Figure 3:**
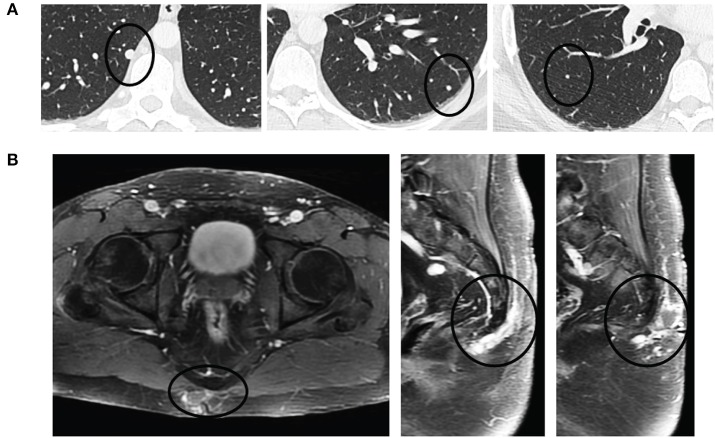
Pulmonary oligometastases upon detection of recurrent sacrococcygeal mass. **(A)** Axial views of the lungs revealing a small, discrete homogeneously hyperdense nodule (black circle) in the right lower lobe (left) that had doubled in size since initial resection (8 mm from 4 mm) and enlarging left lower lobe (middle) and right lower lobe (right) metastases. **(B)** Recurrent post-sacral mass on axial (left) and sagittal (middle, right) fat-saturated contrast-enhanced T1-weighted sequences is heterogeneously enhancing and multilobulated in appearance as it extends through the soft tissues without invasion into the coccyx.

Surveillance imaging with a CT of the chest, abdomen, and pelvis 2 years following resection of the sacrococcygeal mass showed enlargement of the right lower lobe nodule to 8 mm in diameter, and an enlarged left lower lobe nodule measuring 2 mm. Other left and right lower lobe nodules were both stable at 3 mm (Figure 3A). Pelvic imaging revealed new right internal iliac lymphadenopathy with a 10 mm right internal iliac node, previously 6 mm. At the tip of the coccyx was a heterogeneously enhancing lobular mass, measuring 3.5 × 2.1 × 3.1 cm (Figure 3B). There were no destructive bone lesions in the pelvis. A follow-up PET/CT redemonstrated small hypermetabolic nodules in post-sacral soft tissues of the gluteal region. There was no metastatic disease in the abdomen or pelvis. Pelvic lymph nodes were not FDG avid, and the enlarged pulmonary nodules were too small for PET evaluation. The biopsied lung nodule that had enlarged over a 2-year interval displayed similar morphological features to the perisacral tumor, showing large granular tumor cells populated in a fibromyxoid background and strong immunoreactivity for GFAP (Figure 2B), consistent with metastatic MPE (WHO grade I). The patient was referred to an academic center for continued management of the recurrent mass and oligometastatic disease.

**Figure 4:**
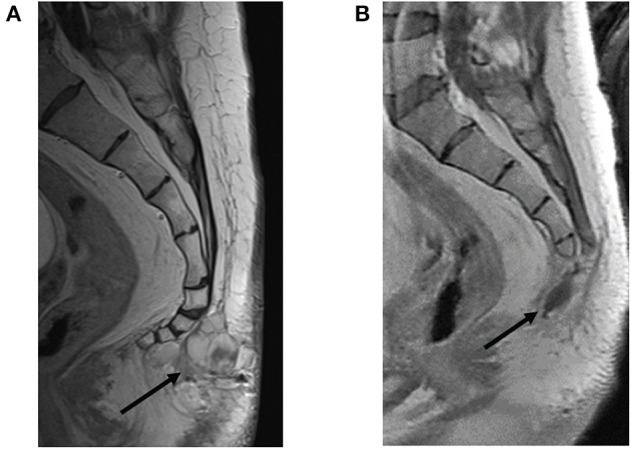
Restaging of recurrent soft tissue mass prior to and following resection. **(A)** Pre-resection sagittal view with contrast-enhanced T1-weighted image showing a multilobulated mass extending from the first through fourth coccygeal segments bordered by a thin plane of fat interposed between the tumor and coccyx without evidence of coccygeal invasion (black arrow). **(B)** Status post-coccygectomy and resection cavity (black arrow) of the previously described associated lobulated mass.

Upon referral to the academic center, staging studies were performed, including a negative brain MRI without any abnormal enhancement and normal flow voids and a normal MRI of the cervical, thoracic, and lumbar spine. MRI of the sacrum revealed two avidly enhancing right internal iliac lymph nodes measuring 14 × 11 mm and 17 × 12 mm. The post-sacral mass was irregular, multilobulated, and enhancing, extending through the soft tissues from the first through fourth coccygeal segments. A thin plane of fat was interposed between the tumor and coccyx without evidence of coccygeal invasion (Figure 4A).

After referral to the academic center's medical oncology, neuro-oncology, and thoracic and orthopedic oncology specialists, both the lung nodule and post-sacral mass were resected with thoracoscopic wedge resection and coccygectomy in the same operative setting. The lung nodule measured 1.1 cm pathologically; it was positive for metastatic tumor, consistent with MPE, with a focus of lymphatic spread. The resected coccygeal mass and pelvic lymph node again demonstrated strong GFAP and S100 expression, and stained negative for brachyury, P40, smooth muscle actin (SMA), calponin, D2-40, and 34bE12. There was weak, focal positivity for pancytokeratin. Both pathologic specimens supported the diagnosis of MPE, and resection margins were negative for tumor in both specimens.

The patient tolerated surgery well. An MRI 5 weeks later showed a stable post-operative resection cavity (Figure 4B). There was minimal enhancement within the right sacrum, as an expected post-operative appearance, and a stable appearance of abnormally enlarged pelvic lymph nodes, inconclusive for known nodal metastases with superimposed reactive post-surgical changes. Of note, this post-operative MRI revealed a very small nodular focus of enhancement along the right aspect of the right S1 nerve root, which in retrospective review, was present and unchanged from prior MRIs, suggestive of stable intrathecal disease. Six weeks following resection, the patient completed a six-week course of 54 Gray (Gy) intensity-modulated radiotherapy (IMRT) to the surgical bed along L4-S3 and involved pelvis. No radiation was administered to the thoracic region. A PET/CT follow up scan 8 weeks following radiation demonstrated expected post-surgical changes, a new hypermetabolic internal iliac chain lymph node, and several small pulmonary nodules in the left lower lobe. The decision was made by the multidisciplinary team of specialists to do surveillance with a re-staging whole-body PET and pelvic MRI in 3-month intervals for the first year. The first of those surveillance scans revealed interval decreased FDG-avidity of the right pelvic wall enlarged lymph node and unchanged small left lower lobe pulmonary nodules. The patient continued with 3-month interval PET/CT plus MRI lumbar/sacrum scans to assess the burden of disease.

## Discussion

An extradural MPE should be considered in the differential of a slow-growing soft tissue mass in the pre- or post-sacral region. The mechanisms by which these ependymal tumors arise in the extradural space are not well-understood, but three mechanisms are thought to be responsible: (1) via direct extension through the dura, as has been characterized from small spinal dysraphism (23), (2) via heterotopic ependymal cells, or (3) via extradural remnants of the filum terminale (24–26). These extradural remnants, known as extramedullary ependymal rests, are vestigial remnants of the coccygeal medullary space (23, 27). Histopathologically, an ependymal rest represents an ependymal-lined cleft and is a derivative of the caudal neural tube (28, 29). Abnormal growth of these ependymal rests can ultimately result in post-sacral MPE (30). Thus, the presence of any lesion near the midline of the spine, particularly in young patients, should alert the clinician to the possibility of a spinal dysraphism or underlying ependymal rest as the etiology for abnormal growth within the extradural tissues.

Extradural MPE have a proclivity to spread most commonly to the lymph nodes, liver, and lungs (19–22). Thus, it is of utmost importance to obtain local control of the primary site with either *en bloc* or gross total resection (GTR) with the addition of early adjuvant radiation therapy (RT). The use of RT should be considered especially if sub-total resection (STR) can only be achieved. Retrospective studies for spinal MPE have found an increased risk of recurrence following STR compared to *en bloc* or GTR (31–33). In cases of compromised capsular integrity with MPE resection, retrospective data suggests that salvage RT could be effective at halting disease progression (32). In one small series of intradural MPE, investigators found that younger patients, those not treated initially with RT, and those with STR had significantly higher rates of tumor recurrence and progression. In this series, patients receiving GTR had a recurrence rate of 10%, whereas those that were removed either piecemeal or in a subtotal fashion had a recurrence rate of 19% (34). Although there is no evidence of a clear dose-response relationship between the amount of radiation and tumor progression, most institutes now recommend radiation doses in the range of 40–50 Gy (31, 35, 36).

The use of adjuvant RT following any extent of resection is being used more commonly now to prevent recurrence. In a study of 35 patients who were treated with surgery and RT, a statistically significant improvement in PFS and local control were observed when compared to those receiving surgery alone (37). A separate multi-institution study of 85 patients with primary MPE showed that post-operative high-dose RT was the only independent predictor of PFS in their study (38). Thus, these studies suggest that RT is both efficacious and safe as a first-line adjuvant therapy for MPE and should be considered regardless of completeness of surgical resection, especially in younger patients.

The advent of molecular profiling for ependymal tumors has dramatically enhanced our understanding and prognostication of specified subgroups. Ependymomas were initially subtyped by histopathologic criteria dividing tumors into grade I subependymomas or MPE, grade II ependymomas, grade II or III ependymoma, RELA fusion-positive, and grade III anaplastic ependymomas (39). Molecular classification for ependymomas has now unveiled a means to distinguish aggressiveness and potential routes for targeted therapies. Pajtler et al. classified 500 ependymal tumors using DNA methylation profiling into nine molecular subgroups (40). Molecular profiling of these subtypes revealed that both spinal MPE (SP-MPE) and spinal ependymomas (SP-EPN) showed a relatively good concordance with the histopathological subtypes MPE (grade I) and ependymoma (grade II) but importantly unveiled that posterior fossa and supratentorial ependymomas often do not correlate with histopathologic grade. In this study, the highest frequency of copy number alteration with loss of 22q was seen in SP-EPN tumors (19 of 21; 90%) as previously described (41–43). However, loss of 22q was not entirely restricted to SP-EPN and was found in a small percentage of SP-MPE. Due to the exceedingly rare presentation of an extradural MPE presented in our case, data is lacking on the molecular characterization of these extradural phenotypes. This methodology, however, would likely yield important information on the behavior of these tumors. We performed next-generation sequencing on the original specimen, which did not reveal 22q loss. Sequencing of this tumor did show a rather low mutational burden and a truncation of *CTNNA1* that codes for alpha-catenin of the cadherin family. Loss of cell adhesion through this gene alteration compromises tumor suppression in epithelial tumors (endometrial carcinoma, colorectal, breast) (44–47) and contributes to cancer cell invasiveness and metastases (48, 49). Given these findings, molecular profiling should be incorporated more often as a methodology for rare tumors such as extradural MPE.

The role of systemic therapy in the treatment of MPE remains largely unknown. There is limited evidence that systemic therapy can be curative. Some studies, however, suggest that tyrosine kinase inhibitors (TKI) can halt disease progression. One case report described the use of imatinib following temozolomide (TMZ) failure based on the tumor's positivity for platelet-derived growth factor receptor, and the patient remained without disease progression for 11 months (50). Another study reported the use of sorafenib for three intrathoracic MPE metastases detected 20 years after initial coccygeal resection resulting in disease stabilization, with acceptable quality of life, for 1 year prior to progression of disease (51).

Long-term survival data for spinal MPE suggests that treatment of the primary site with surgery and adjuvant radiation offer the best rates of local control and PFS (32). However, these outcomes are based on a combination of retrospective studies of spinal, not extradural MPE. Furthermore, all of these studies have failed to detect a significant alteration in patient overall survival (OS) with the inclusion of adjuvant RT (32, 33). Retrospective data for spinal MPE suggests that OS is more so influenced by initial extent of resection (31, 32). There also remains insufficient evidence on the role of RT to oligometastatic foci in providing any further survival benefit or extending time to recurrence for MPE. Thus, prospective studies assessing the role of upfront treatment of oligometastases with local resection ± adjuvant RT are heavily needed for improved understanding of the natural disease of extradural MPE.

## Conclusion

Extradural MPE, although rare in incidence, have a common tendency to spread to the lymph nodes, liver, and lung. In the current case, a slow growing and rather indolent extradural MPE warranted invasive intervention when it expanded in size, the time at which this tumor likely spread to the lungs. From case report experience, one critical early intervention for control of this tumor is primary resection and early adjuvant radiation. The primary site was treated with surgery, of which the extent of the initial resection was unfortunately unknown. Molecular sequencing on the original tumor revealed a low mutational burden but showed a loss of alpha-catenin expression potentially favoring a greater metastatic potential. Within 2 years, the primary tumor recurred with a more indicative presentation of both nodal and pulmonary metastases. This begs the question if the natural history of disease recurrence can be altered by adjuvant radiation to the primary site. In this case upon disease recurrence, the decision was made to treat with GTR of the oligometastatic focus in the lungs along with GTR and adjuvant IMRT to the pelvis. Post-treatment monitoring is not well-defined for extradural metastatic MPE. Clinicians encountering this situation agree that certainly frequent and prolonged post-operative follow up is necessary, as late recurrences can occur several years after the primary tumor is removed. For this reason, the case reported here will be monitored frequently with pelvic scans and whole-body PET, given the proclivity of this tumor to spread to extradural sites. The manner in which this rare type of MPE presented reflects how distinguishable these extradural MPE may behave. As such, this unique case highlights the multi-disciplinary approach required to manage both primary site and oligometastases by neuro-oncology and medical oncology communities.

## Ethics Statement

A written informed consent was obtained from the patient for the publication of this case report.

## Author Contributions

KP, RR, BT, WE, and JK were involved in patient management with regards to initial presentation, diagnosis, treatment, and follow-up. BT and WE assisted with obtaining the subsequent tissue diagnoses. RM, PP, and CT contributed to the histopathologic diagnosis, immunohistochemistry, and secondary review of all specimens presented. KB collected and interpreted the patient data. KB, KP, and RR wrote the manuscript. All authors read and approved the final manuscript.

### Conflict of Interest

The authors declare that the research was conducted in the absence of any commercial or financial relationships that could be construed as a potential conflict of interest.

## Abbreviations

CNS: central nervous system
CSF: cerebrospinal fluid
CT: computed tomography
EMA: epithelial membrane antigen
FDG: fluorodeoxyglucose
GFAP: glial fibrillary acidic protein
Gy: gray
GTR: gross total resection
IMRT: intensity-modulated radiation therapy
MRI: magnetic resonance imaging
MPE: myxopapillary ependymoma
OS: overall survival
PET: positron emission tomography
PFS: progression-free survival
RT: radiation therapy
SMA: smooth muscle actin
SP-EPN: spinal ependymoma
SP-MPE: spinal myxopapillary ependymoma
STR: sub-total resection
TMZ: temozolomide
TKI: tyrosine kinase inhibitor
VATS: video-assisted thoracic surgery.
